# Supplementary material for: Hybrid Models and Biological Model Reduction with PyDSTool
Source: PLoS Comput Biol. 2012 Aug 9;8(8):e1002628. doi: 10.1371/journal.pcbi.1002628 (PMC3415397; doi:10.1371/journal.pcbi.1002628)
Supplement: Text S4 — Complete source code for the PyDSTool package (version 0.88.120504). Includes API documentation and help files linking to web pages. This file is identical to the current public release on Sourceforge.net. (ZIP) [file pcbi.1002628.s004.zip › PyDSTool/html/PyDSTool.Toolbox.phaseplane.nullcline-class.html]

xml version="1.0" encoding="ascii"?


PyDSTool.Toolbox.phaseplane.nullcline


| Home | Trees | Indices | Help | PyDSTool |
| --- | --- | --- | --- | --- |

Package PyDSTool ::
Package Toolbox ::
Module phaseplane ::
Class nullcline

[hide private]

[frames] | no frames]

# Class nullcline

source code

```
object --+
         |
        nullcline
```

---

```
Nullcline representation class in 2D (x, y) plane parameterizable
by x variable only. A third-order univariate spline will be fitted to
the given sample point array using all as knots, and available
through the 'spline' attribute.

Input:
  Names are given to the x and y axis directions at initialization.
  Third argument nullc_array is the N-by-2 array of N two-dimensional
    data points.
  The final, optional argument x_relative_scale indicates the relative
    scale of the x values' domain compared to the y values. This is
    used to provide a form of subjective normalization for measuring
    curvature and other geometric properties when plotting y vs. x
    on these scales. E.g if x values range over -50 to +50, while
    y ranges over 0 to 1, x_relative_scale=100 is appropriate.

N.B.: Only works for nullclines that can be written y = f(x). Future
versions may support non-functions.

Some functions acting on nullclines currently also rely on monotonicity
of y=f(x), and will verify this using the is_monotonic method before
proceeding. Future versions will attempt to resolve that.
```


|  |  |
| --- | --- |
| Instance Methods | [hide private] |

|  |  |
| --- | --- |
| \_\_init\_\_(self, xname, yname, nullc\_array, x\_relative\_scale=1)  x.\_\_init\_\_(...) initializes x; see x.\_\_class\_\_.\_\_doc\_\_ for signature | source code |

|  |  |
| --- | --- |
| \_\_call\_\_(self, x)  Returns a scalar if x is scalar. | source code |

|  |  |
| --- | --- |
| createParameterization(self)  Creates a (non-arc-length) parameterization | source code |

|  |  |
| --- | --- |
| toPointset(self, with\_param=False) | source code |

|  |  |
| --- | --- |
| sample\_x\_domain(self, refine=0) | source code |

|  |  |
| --- | --- |
| tgt\_vec(self, x, rescale=False)  Return tangent vector to an interior point of nullcline, normalized to length 1. | source code |

|  |  |
| --- | --- |
| curvature(self, xdata)  Signed curvature at x is returned (scalar) for scalar x, otherwise array of such scalars for 1D array x. | source code |

|  |  |
| --- | --- |
| grad\_curvature(self, xdata)  Derivative of the curvature at x is returned (scalar) for scalar x, otherwise array of such scalars for 1D array x. | source code |

|  |  |
| --- | --- |
| curvature\_at\_sample\_points(self, refine=0)  Returns array of signed scalars for each x data point (knot) of the nullcline spline (computed just inside endpoints by 1% of distance to next innermost sample point). | source code |

|  |  |
| --- | --- |
| grad\_curvature\_at\_sample\_points(self, refine=0)  Returns array of signed scalars for each x data point (knot) of the nullcline spline (computed just inside endpoints by 1% of distance to next innermost sample point). | source code |

|  |  |
| --- | --- |
| concavity(self, xdata)  Concavity scalar +/- 1 or 0 at x is returned for scalar x, otherwise array of such scalars for 1D array x. | source code |

|  |  |
| --- | --- |
| concavity\_at\_sample\_points(self, refine=0)  Returns array of +/- 1 or 0 scalars for each x data point (knot) of the nullcline spline (computed just inside endpoints by 1% of distance to next innermost sample point). | source code |

|  |  |
| --- | --- |
| crop(self, xdom, ydom, resample\_frac=0.1)  Returns a new Nullcline object cropped to the (x, y) domain given by pairs xdom and ydom. | source code |

|  |  |
| --- | --- |
| is\_monotonic(self) | source code |

|  |  |
| --- | --- |
| \_\_copy\_\_(self) | source code |

**Inherited from `object`**:
`__delattr__`,
`__getattribute__`,
`__hash__`,
`__new__`,
`__reduce__`,
`__reduce_ex__`,
`__repr__`,
`__setattr__`,
`__str__`


|  |  |
| --- | --- |
| Properties | [hide private] |

**Inherited from `object`**:
`__class__`


|  |  |
| --- | --- |
| Method Details | [hide private] |

|  |  |
| --- | --- |
| \_\_init\_\_(self, xname, yname, nullc\_array, x\_relative\_scale=1)  *(Constructor)* | source code |

x.\_\_init\_\_(...) initializes x; see x.\_\_class\_\_.\_\_doc\_\_ for
signature

Overrides: object.\_\_init\_\_: *(inherited documentation)*

|  |  |
| --- | --- |
| tgt\_vec(self, x, rescale=False) | source code |

Return tangent vector to an interior point of nullcline, normalized to
length 1. Set rescale option (default False) to return a vector rescaled
according to relative x vs. y scales (thus it's no longer a tangent
vector in that case).

|  |  |
| --- | --- |
| curvature(self, xdata) | source code |

Signed curvature at x is returned (scalar) for scalar x, otherwise
array of such scalars for 1D array x.

Positive values mean concave "up" in the plane.

|  |  |
| --- | --- |
| curvature\_at\_sample\_points(self, refine=0) | source code |

Returns array of signed scalars for each x data point (knot) of the
nullcline spline (computed just inside endpoints by 1% of distance to
next innermost sample point).

Positive values mean concave "up" in the plane. Assumes
nullcline is monotonic.

|  |  |
| --- | --- |
| grad\_curvature\_at\_sample\_points(self, refine=0) | source code |

Returns array of signed scalars for each x data point (knot) of the
nullcline spline (computed just inside endpoints by 1% of distance to
next innermost sample point).

Assumes nullcline is monotonic.

|  |  |
| --- | --- |
| concavity(self, xdata) | source code |

Concavity scalar +/- 1 or 0 at x is returned for scalar x, otherwise
array of such scalars for 1D array x.

Positive values mean concave "up" in the plane.

|  |  |
| --- | --- |
| concavity\_at\_sample\_points(self, refine=0) | source code |

Returns array of +/- 1 or 0 scalars for each x data point (knot) of
the nullcline spline (computed just inside endpoints by 1% of distance to
next innermost sample point).

Positive values mean concave "up" in the plane. Assumes
nullcline is monotonic.

|  |  |
| --- | --- |
| crop(self, xdom, ydom, resample\_frac=0.1) | source code |

Returns a new Nullcline object cropped to the (x, y) domain given by
pairs xdom and ydom.

If resample\_frac > 0 (default 0.1), points at the domain ends and
additional sample points will be included in the new nullcline object
regardless of whether they were in the original sample point set for the
cropped nullcline. New points will be selected at the rate indicated,
measured as a fraction of the domain width. Points that are as close as
1/10th of this rate to existing sample points will be excluded, to avoid
ill-conditioning the spline shape in the resampling.

Given the assumption that nullclines are montonic functions of x, this
guarantees a unique, contiguous piece of the nullcline is returned.

  


| Home | Trees | Indices | Help | PyDSTool |
| --- | --- | --- | --- | --- |

|  |  |
| --- | --- |
| Generated by Epydoc 3.0.1 on Fri May 4 15:24:09 2012 | http://epydoc.sourceforge.net |
